# Highlight—Untangling the Genetic Basis of Sociality in Spiders

**DOI:** 10.1093/gbe/evaa035

**Published:** 2020-03-31

**Authors:** Casey McGrath

The idea of a complex spider society—in which thousands of spiders live, hunt, and raise their young together in a single colony—is unsettling to many of us. We are perhaps lucky then that this scene is relatively rare among arachnids. Among the 40,000 known species of spiders, the vast majority live solitary lives and will often show aggression toward other spiders they encounter, even within their own species. There are <25 known species of social spiders, distributed broadly across six different families and nine different genera. Not only do these spiders live in social groups, but also they produce populations that grow over time as new offspring are added to the nest, enabling the capture of increasingly large prey as the colony expands, and even give rise to new daughter colonies. As social creatures ourselves, humans have long been interested in the evolutionary innovations that enable social cooperation. In a new article in *Genome Biology and Evolution* titled “Comparative genomics identifies putative signatures of sociality in spiders,” researchers provide one of the first glimpses into the genetic underpinnings for how a solitary spider evolves into a social one.

**Fig. 1.**
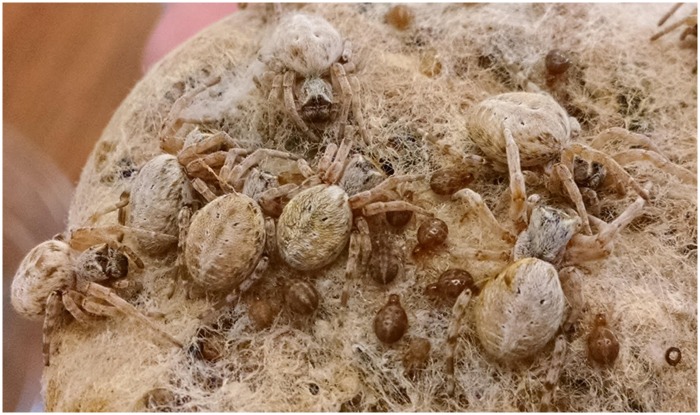
—A colony of the social spider *Stegodyphus dumicola*. (Photo credit: Noa Pinter-Wollman.)

The research, led by Dr Chao Tong, a postdoc in the lab of Dr Timothy Linksvayer at the University of Pennsylvania, represents one of the first comparative genomic studies to be conducted in spiders (Tong et al. 2020). According to Dr Tong, “The high complexity and large size of spider genomes has constrained the development of genomic resources for spiders.” Because of this, earlier studies compared individual spider genomes to insect genomes and sought mainly to identify venom and silk genes. In the new study, however, a curiosity about the genetic basis of social life led Dr Tong and his colleagues to compare the genomes of seven spider species: two social species in the genus *Stegodyphus* that evolved sociality independently, and five solitary species in the genera *Parasteatoda, Acanthoscurria, Nephila, Loxosceles*, and *Latrodectus* (**fig. 1**).

The analysis revealed a number of interesting findings. First, rapidly evolving genes in the two social species were involved not only in behavior but also in immunity, indicating that group living may require better defenses against pathogens that spread more easily in dense social groups. In contrast, genes that were rapidly evolving in the solitary species were enriched for energy metabolism processes. Dr Tong notes that this is the opposite pattern from what is observed in social insects such as bees and ants, in which metabolic genes evolve more rapidly. Still, in both insects and spiders, there appear to be metabolic differences between solitary and social organisms that may reflect differences in hunting and feeding behaviors.

In addition, the researchers found that the genomes of the two social spiders exhibited a higher rate of evolution overall than those of the solitary species. Although this might reflect a greater number of genes under positive selection, the authors are quick to point out that this pattern may also stem from demographic features that characterize social spiders, such as a female-skewed sex ratio (social spiders have more female offspring than males) and high levels of inbreeding.

Perhaps most interesting of all, the new study identified a set of rapidly evolving genes that showed brain-specific expression and were enriched for social behavioral processes. These genes represent top candidates for those that influence social behavior and may have been involved in the evolution of spider sociality.

The researchers note an important limitation of their analysis: since the two social spiders they studied are from the same genus and they did not have access to a solitary spider from this genus, it is difficult to untangle which patterns are directly related to sociality and which may simply characterize spiders in the genus *Stegodyphus*—both social and nonsocial species. Thus, the group has already started their next comparative genomics project to verify the above patterns in a larger set of species, including more social spiders and their solitary relatives within the same genus. Realizing that the success of this endeavor may require establishing new collaborations due to the scarcity of spider genomic resources, Dr Tong would like to put out a call to other researchers: “We welcome samples from other researchers, as we want to include as many spiders as possible in future studies, including social, subsocial, and solitary spider samples from multiple genera.”
